# Supplementary material for: Zika Virus-Infected Monocyte Exosomes Mediate Cell-to-Cell Viral Transmission
Source: Cells. 2024 Jan 12;13(2):144. doi: 10.3390/cells13020144 (PMC10814160; doi:10.3390/cells13020144)
Supplement: Supplementary file 1 [file cells-13-00144-s001.zip › cells-2749888-supplementary.pdf]

Zika Virus-Infected Monocyte Exosomes Mediate Cell-to-Cell Viral Transmission

Supplementary Materials

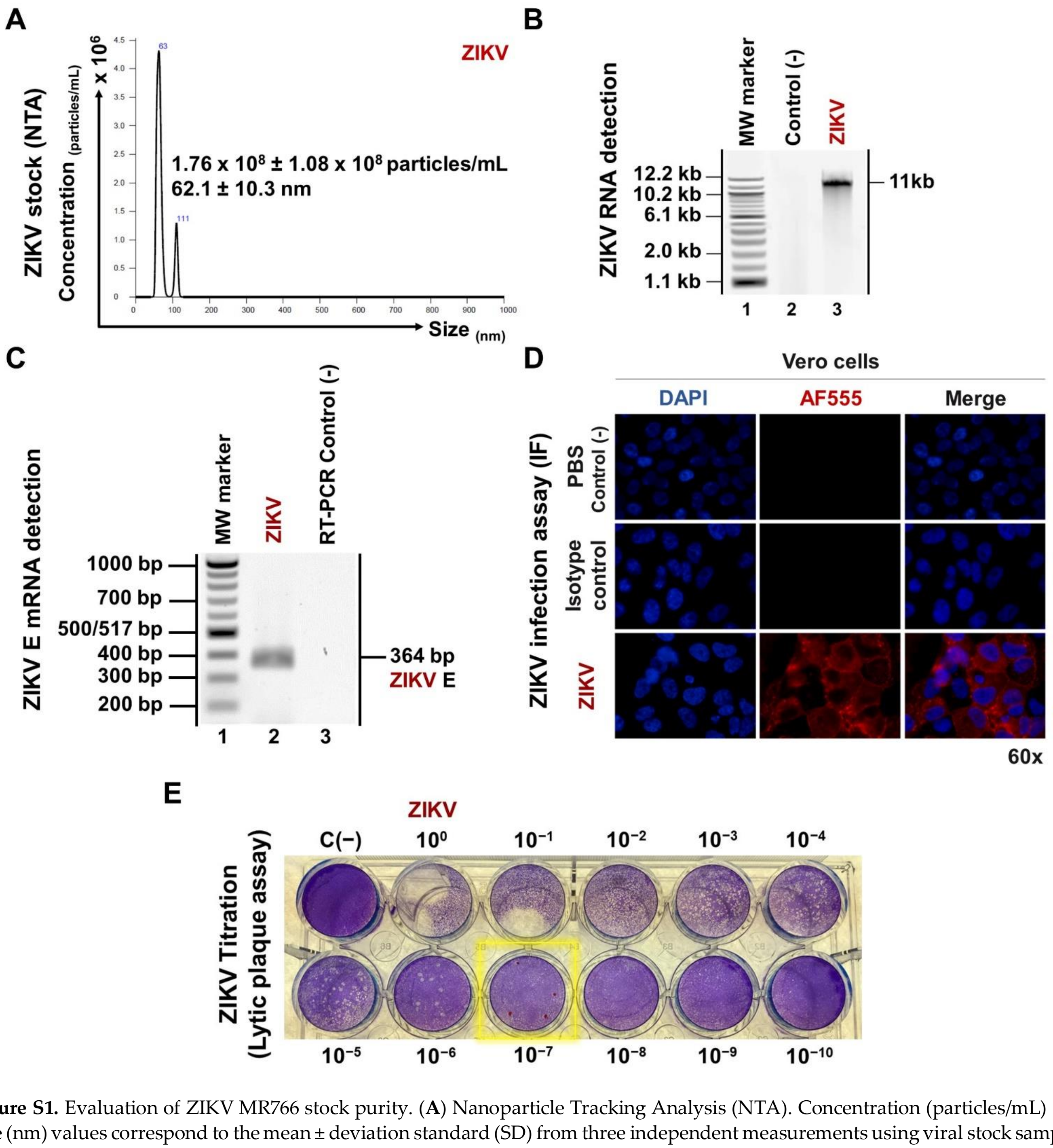

Figure S1. Evaluation of ZIKV MR766 stock purity. (**A**) Nanoparticle Tracking Analysis (NTA). Concentration (particles/mL) and size (nm) values correspond to the mean ± deviation standard (SD) from three independent measurements using viral stock samples diluted at 1:50. (**B**) Detection of total viral RNA from purified ZIKV stock sample by electrophoresis on 2% ethidium bromide-stained 1.2% agarose gel. (**C**) Amplification (RT-PCR) of ZIKV RNA in purified ZIKV stock sample. The ZIKV amplicon (364 bp from the E genome conserved region) was visualized on 2% ethidium bromide-stained 1.2% agarose gel. (**D**) Detection of the ZIKV E protein (red) by fluorescence microscopy (60×) in Vero cells. (**E**) Purified ZIKV stock titration by lytic plaque assay. The viral titer (1.0×10^8 PFU/mL), expressed as plaque-forming units (PFU) per milliliter (mL), was calculated with the formula: PFU/mL = N/(V × D), where *N* corresponds to the counted plaques average number, *V* to the dilution volume inoculated, and *D* to the less concentrated dilution where plaques were counted.

Table S1. Antibodies used for detection of viral (E and NS1) proteins and cell markers (CD14, CD16, CD11b, CD11c, CD80, CD86, CD63, CD81, TSG101, and Alix) by FACS/IF.

Antibody	Catalog #	Manufacturer
Mouse anti-ZIKV ENV IgG1 antibody (identified as anti-E ZIKV) clone 1413267.	CABT-B8528	CD Creative Diagnostics, New York, NY, USA
Mouse anti-ZIKV NS1 IgG1 antibody clone E107.	MA5-24585	Invitrogen, ThermoFisher Scientific, Waltham, MA, USA
Phycoerythrin (PE)-conjugated mouse anti-human CD14 IgG1 antibody clone HCD14	325606	BioLegend, San Diego, CA, USA
Mouse anti-CD16 IgG1 antibody.	555404	BD Pharmingen, BD Biosciences, San Jose, CA, USA
PE-conjugated mouse anti-human CD11b IgG1 antibody clone ICRF44.	301306	BioLegend
PE-conjugated mouse anti-human CD11c IgG1 antibody clone 3.9.	301605	BioLegend
Fluorescein isothiocyanate (FITC)-conjugated mouse anti-CD80 human IgG1 antibody clone 2D10.	305205	BioLegend
PE-conjugated mouse anti-human CD86 IgG2b antibody clone IT2.2.	305405	BioLegend
PE-conjugated mouse anti-human CD63 IgG1 antibody.	557305	BD Pharmingen
PE-conjugated mouse anti-human CD81 IgG1 antibody clone 1D6.	566714	BD Pharmingen
Mouse anti-human TSG101 IgG2a antibody.	sc-7964	Santa Cruz Biotechnology, Dallas, TX, USA
Mouse anti-human Alix (AIP1) IgG2a antibody.	sc-365921	Santa Cruz Biotechnology
Alexa Fluor 555-conjugated donkey anti-mouse IgG secondary antibody.	A-31570	Thermo Fisher Scientific
PE-conjugated mouse IgG1 antibody clone MOPC-21 (Isotype control).	400112	BioLegend
FITC-conjugated mouse IgG1 antibody clone MOPC-21 (Isotype control).	400107	BioLegend
PE-conjugated mouse IgG2b antibody clone MPC-11 (Isotype control).	400313	BioLegend
Mouse IgG1 antibody clone P3.6.2.8.1 (Isotype control).	14-4714-82	eBioscience, San Diego, CA, USA
Mouse IgG2a antibody clone MG2a-53 (Isotype control).	401501	BioLegend

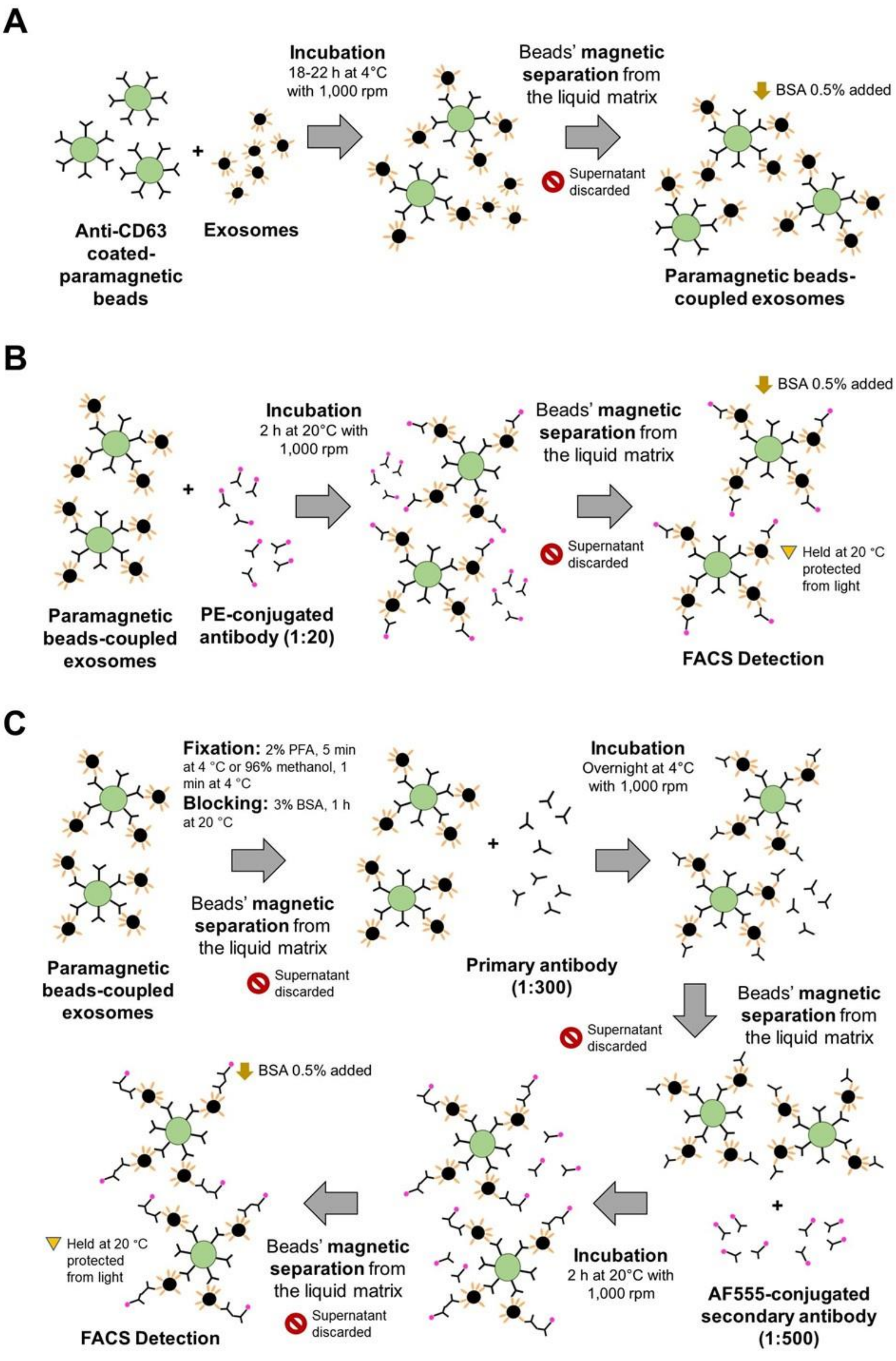

Figure S2. Paramagnetic-bead coupling for the identification of exosomal markers and viral components in exosome isolates by FACS (graphic description). (**A**) Exosome coupling to paramagnetic beads. (**B**) Detection of CD63 and CD81 in paramagnetic-bead-coupled exosomes. (**C**) Detection of TSG101, Alix, and viral components (E and NS1 proteins) in paramagnetic-bead-coupled exosomes.

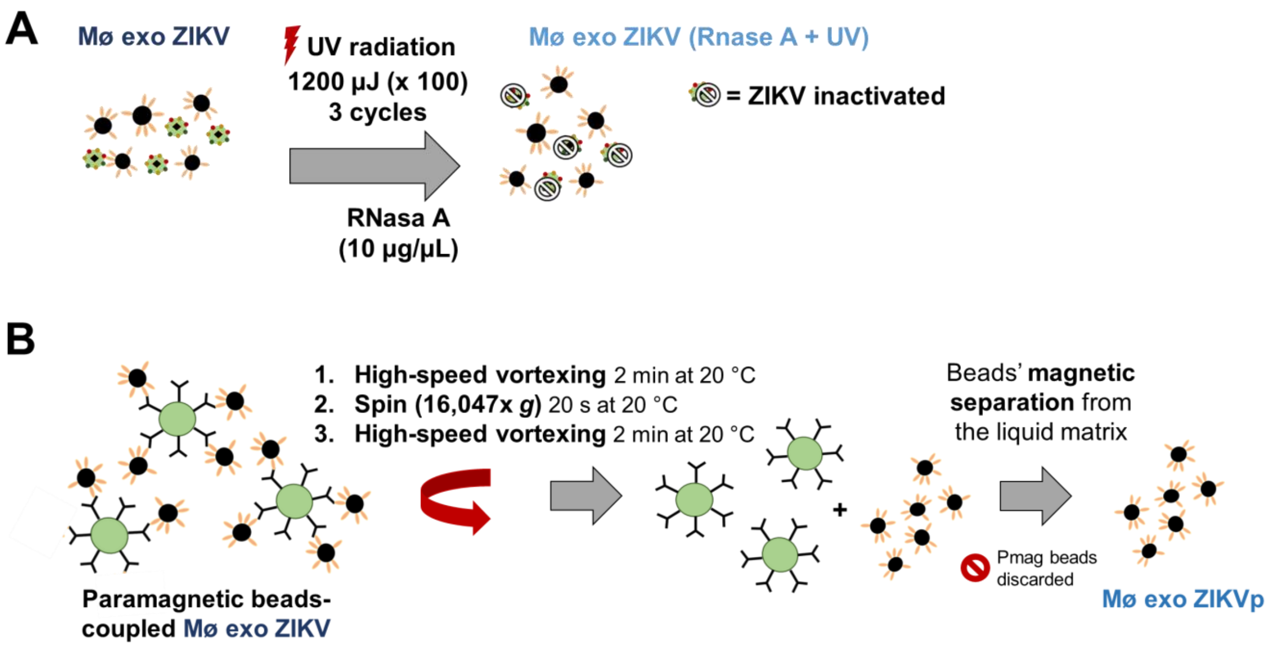

Figure S3. ZIKV inactivation/Exosome purification (graphic description). (**A**) UV irradiation and RNase A assay for ZIKV inactivation in Mø exo ZIKV isolates. (**B**) Mø exo ZIKV uncoupling from anti-CD63-antibody-coated paramagnetic beads to obtain purified exosomes (Mø exo ZIKVp).

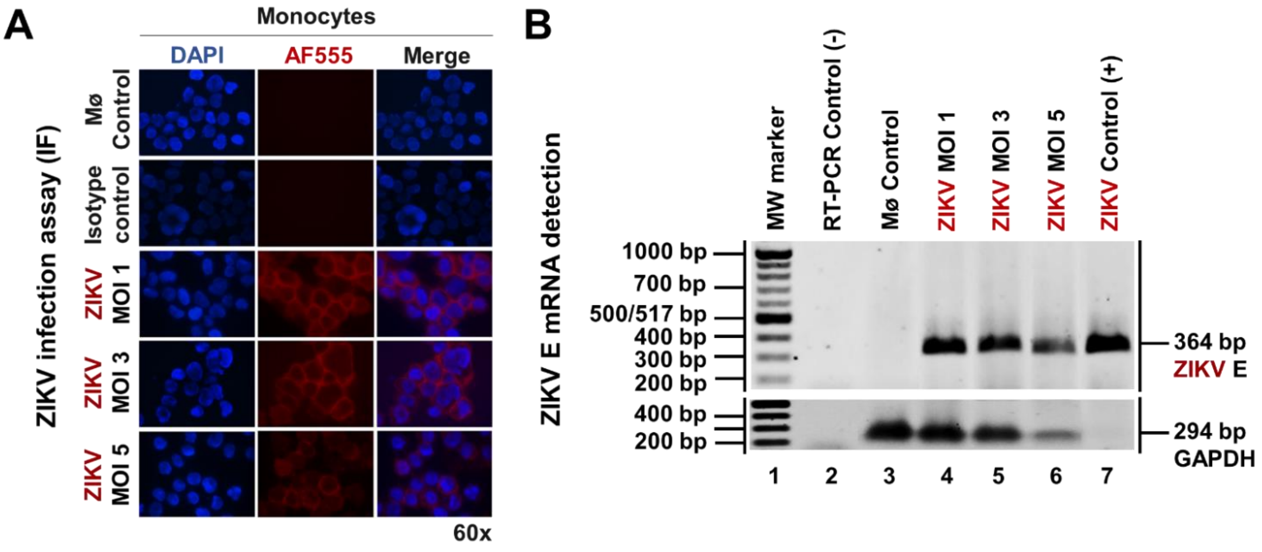

Figure S4. ZIKV (MOI 1, 3, and 5) infection assays in human monocytes. (**A**) Detection of ZIKV E protein (red) by fluorescence microscopy (60×) in ZIKV-infected monocytes at 24 h p.i. (**B**) Detection (RT-PCR) of ZIKV mRNA transcripts in ZIKV-infected monocytes at 24 h p.i. The ZIKV amplicon (364 bp from the E genome conserved region) was visualized on 2% ethidium bromide-stained 1.2% agarose gel.

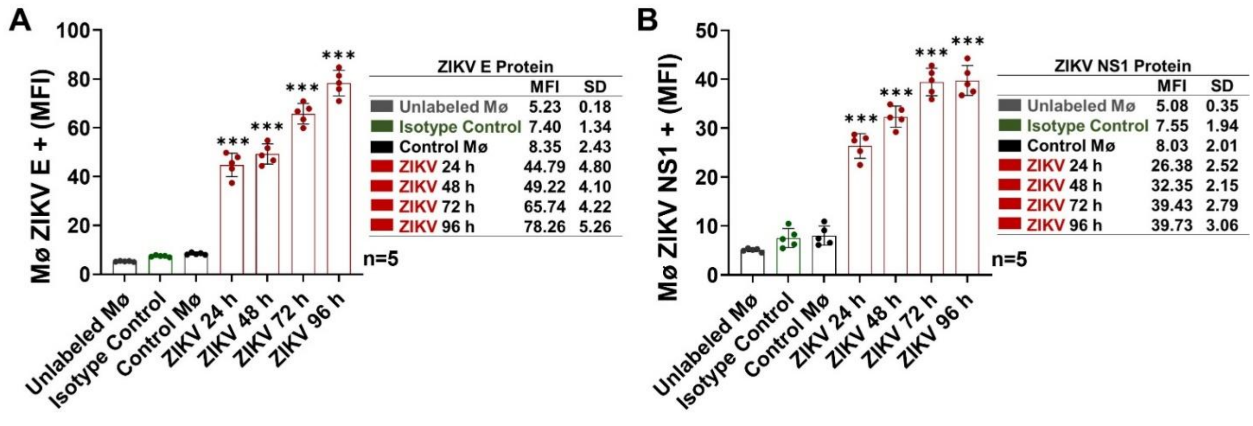

Figure S5. Citofluorometry mean fluorescence intensity (MFI) values from ZIKV MOI 1-monocyte (Mø) infection curve. (**A**) Detection of the ZIKV E protein at 24, 48, 72, and 96 h p.i. (**B**) Detection of the ZIKV NS1 protein at 24, 48, 72, and 96 h p.i. The MFI values of Mø positive for viral E or NS1 proteins were compared with the Control Mø by one-way ANOVA. Statistical significance is denoted as *** when $p < 0.0001$. Unlabeled Mø (gray), Isotype Control (green), Control Mø (black), and ZIKV MOI 1 (red).

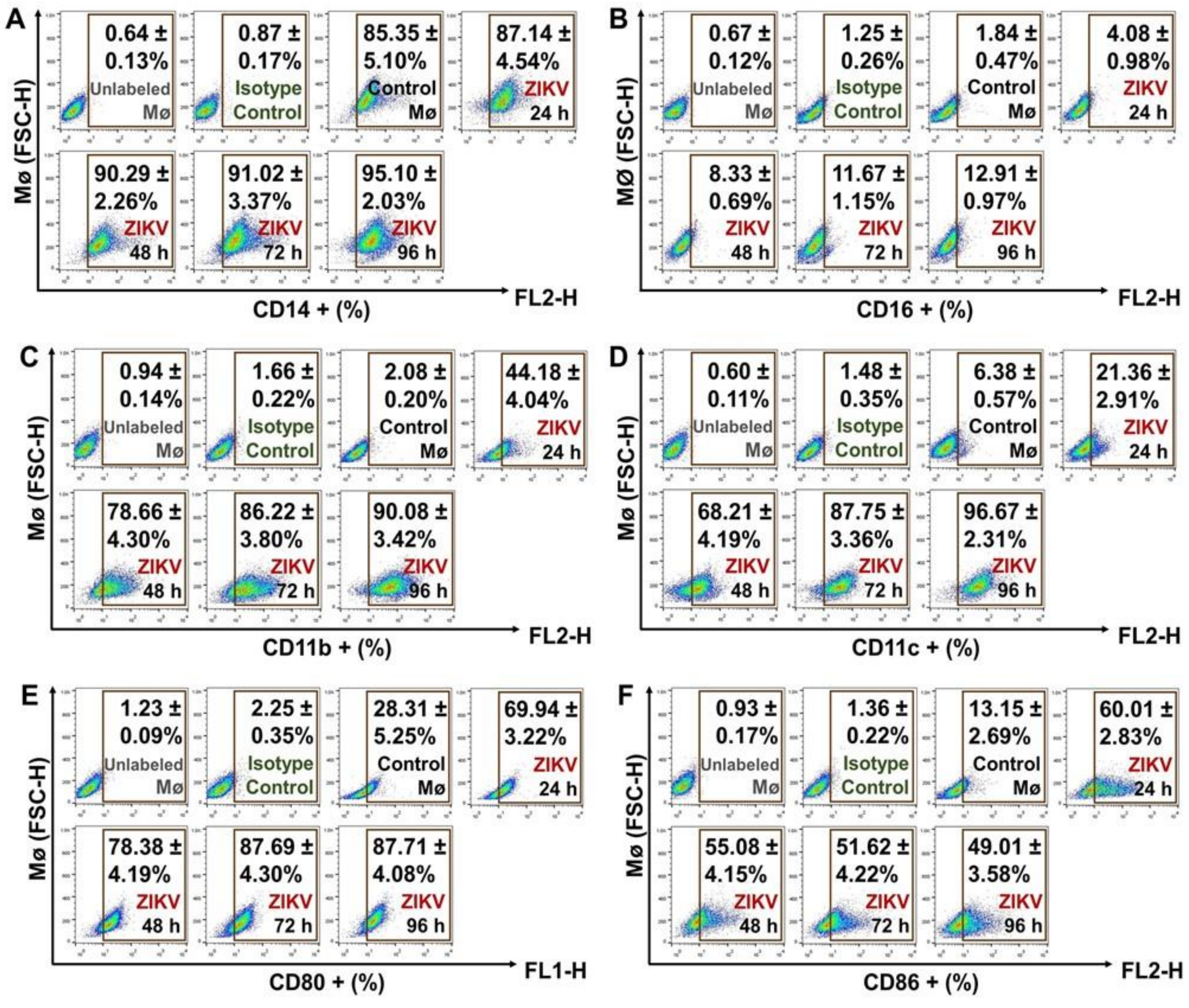

Figure S6. FACS detection curves for monocyte (Mø) membrane marker (representative dot plots). (**A**) Percentages of Mø positive for CD14. (**B**) Percentages of Mø positive for CD16. (**C**) Percentages of Mø positive for CD11b. (**D**) Percentages of Mø positive for CD11c. (**E**) Percentages of Mø positive for CD80. (**F**) Percentages of Mø positive for CD86. Dot plots are the representative mean ± standard deviation (SD) from five independent experiments.

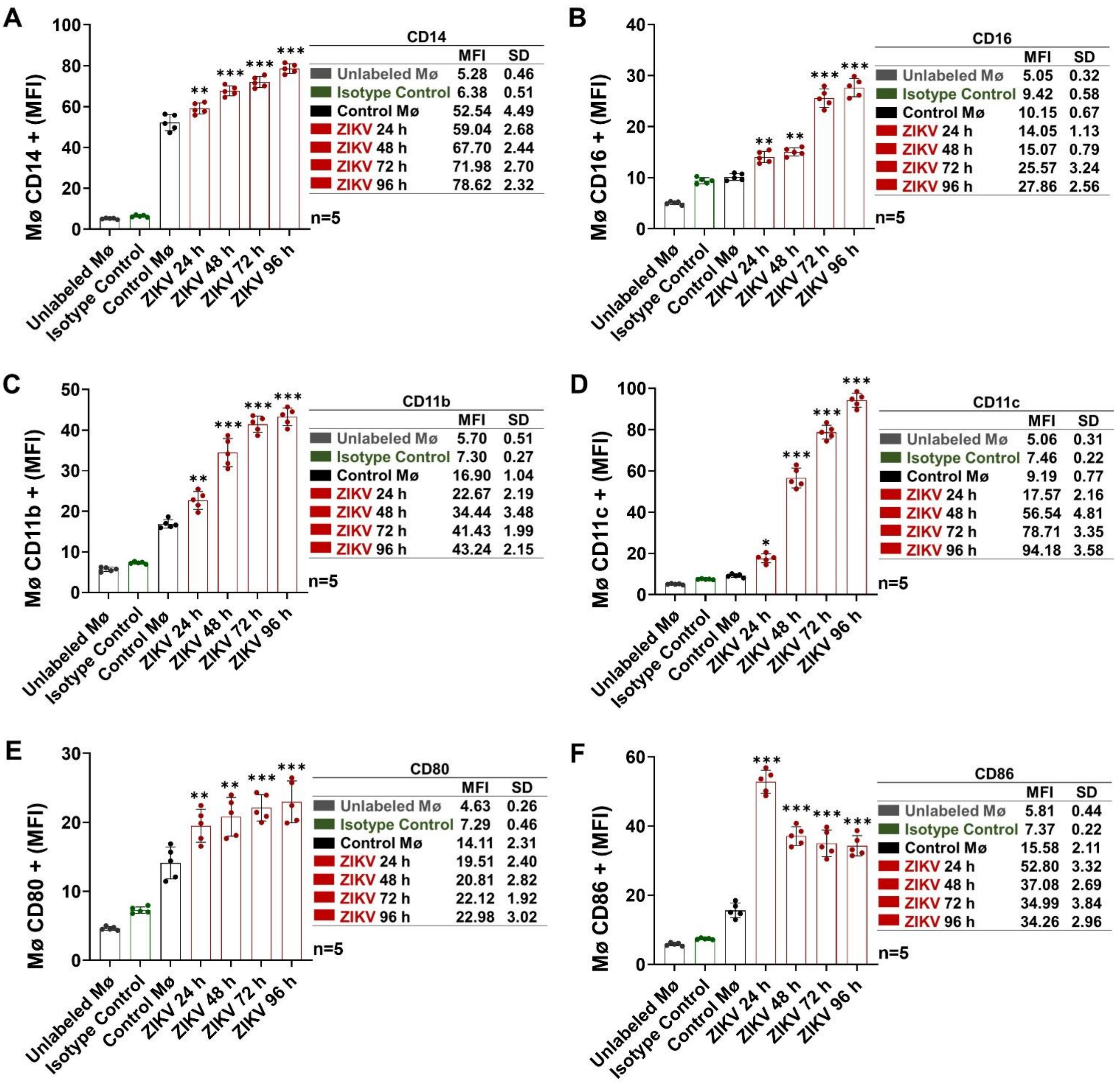

Figure S7. Citofluorometry MFI values from detection of cell markers at 24, 48, 72, and 96 h p.i. in ZIKV-infected Mø. (**A**) MFI values of Mø positive for CD14. (**B**) MFI values of Mø positive for CD16. (**C**) MFI values of Mø positive for CD11b. (**D**) MFI values of Mø positive for CD11c. (**E**) MFI values of Mø positive for CD80. (**F**) MFI values of Mø positive for CD86. The MFI values of cells positive for CD14, CD16, CD11b, CD11c, CD80, and CD86 were compared with the Control Mø values by one-way ANOVA. Statistical significance is denoted as follows: * when $p < 0.05$, ** when $p < 0.01$, and *** when $p < 0.0001$.

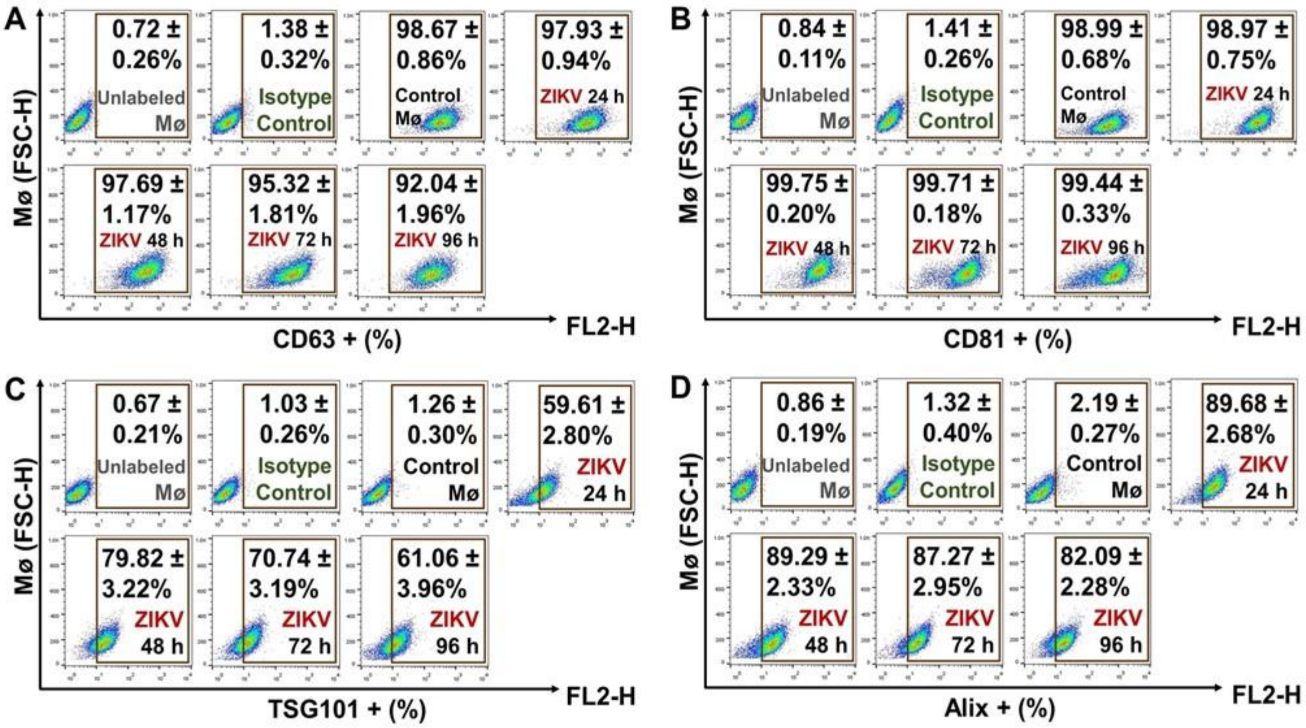

Figure S8. FACS detection curves for monocyte (Mø) membrane endosomal-trafficking-associated proteins (representative dot plots). (**A**) Percentages of Mø positive for CD63. (**B**) Percentages of Mø positive for CD81. (**C**) Percentages of Mø positive for TSG101. (**D**) Percentages of Mø positive for Alix. Dot plots are the representative mean ± SD from five independent experiments.

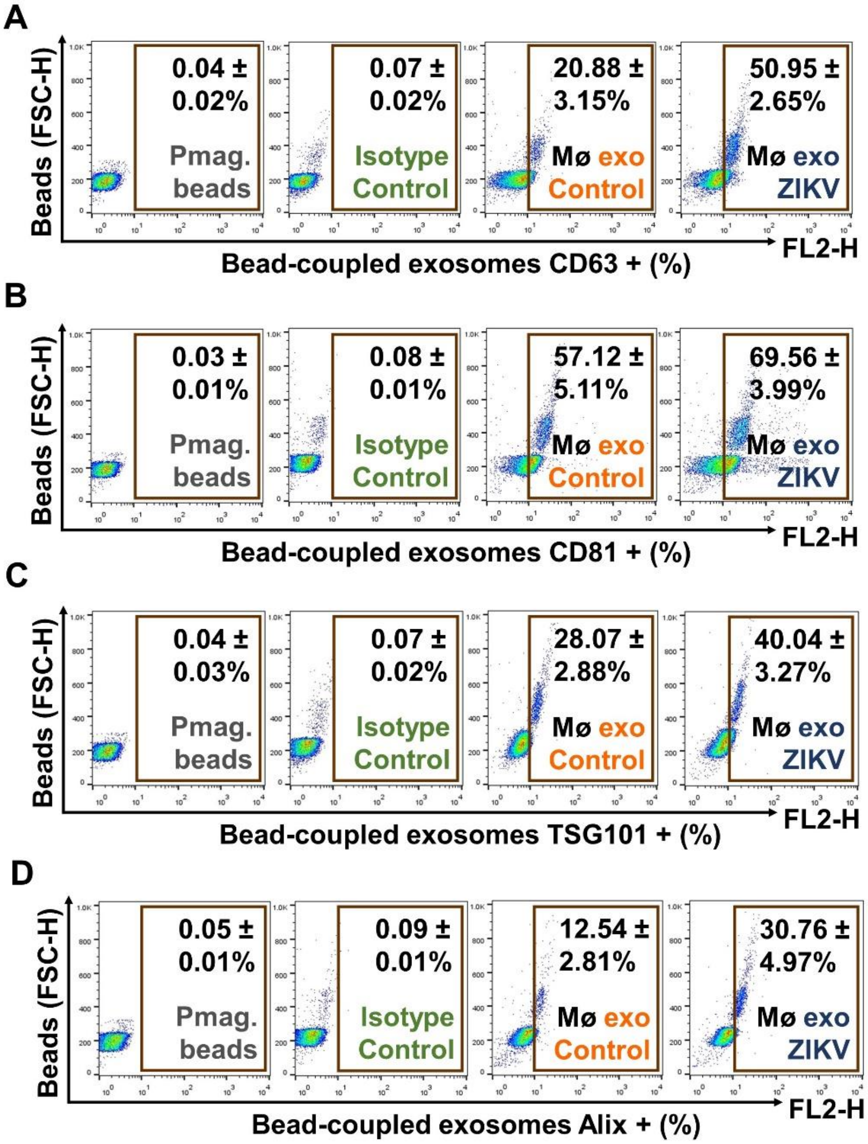

Figure S9. Detection of exosome markers (representative dot plots) by FACS. (**A**) Pmag-bead-coupled exosomes CD63+ percentages. (**B**) Pmag-bead-coupled exosomes CD81+ percentages. (**C**) Pmag-bead-coupled exosomes TSG101+ percentages. (**D**) Pmag-bead-coupled exosomes Alix+ percentages. Dot plots are the representative mean ± SD from five independent experiments.

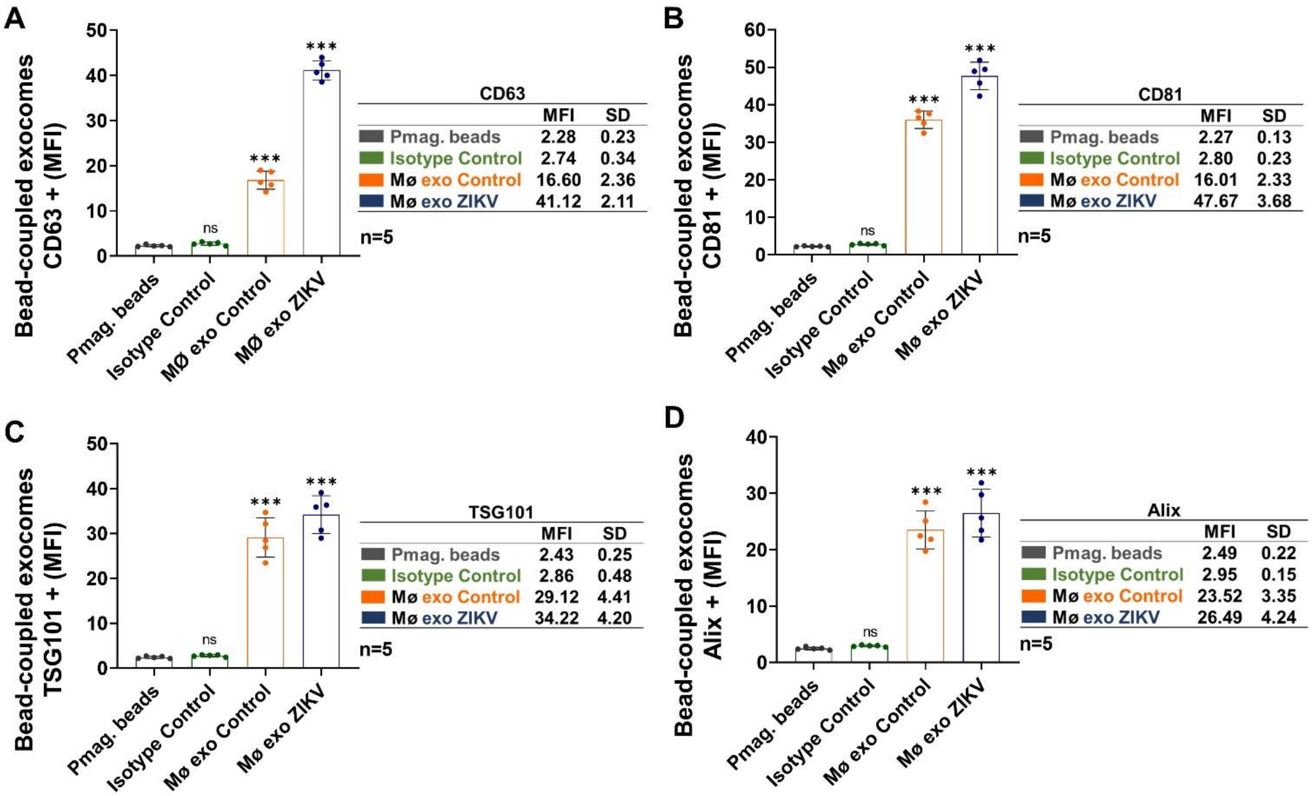

Figure S10. Citofluorometry MFI values from detection of exosome markers. (**A**) MFI values of pmag-bead-coupled exosomes CD63+. (**B**) MFI values of pmag-bead-coupled exosomes CD81+. (**C**) MFI values of pmag-bead-coupled exosomes TSG101+. (**D**) MFI values of pmag bead-coupled exosomes Alix+. The MFI values of CD63, CD81, TSG101, and Alix from pmag-bead-coupled exosomes were compared with the pmag beads values by one-way ANOVA. Statistical significance is denoted as *** when p < 0.0001. ns = no significance. Pmag-beads (gray), Isotype control (green), Mø exo Control (orange), and Mø exo ZIKV (dark blue).

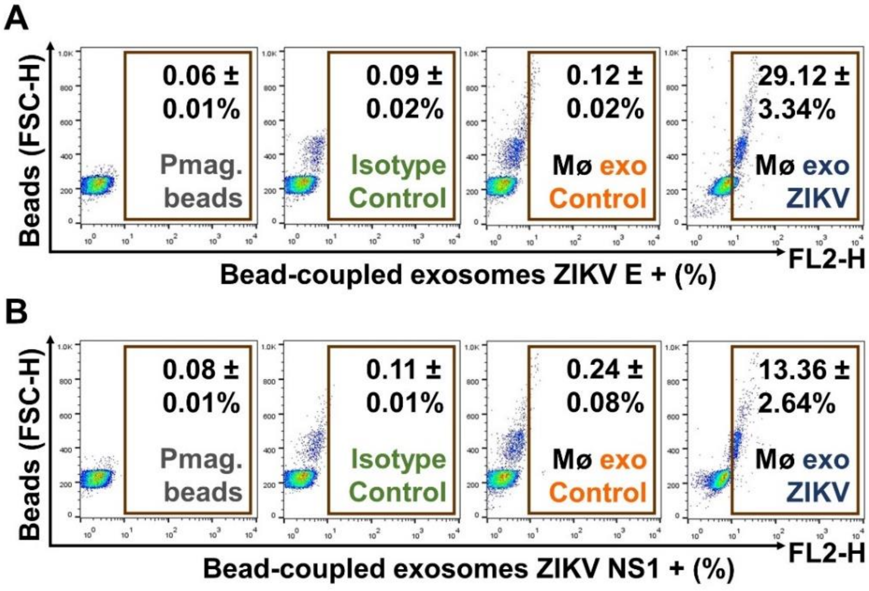

Figure S11. Detection of viral E and NS1 proteins in pmag-bead-coupled exosome isolates (representative dot plots) by FACS. (**A**) Percentages of pmag-bead-coupled exosomes positive for ZIKV E protein. (**B**) Percentages of pmag-bead-coupled exosomes positive for ZIKV NS1 protein. Dot plots are the representative mean ± SD from five independent experiments.

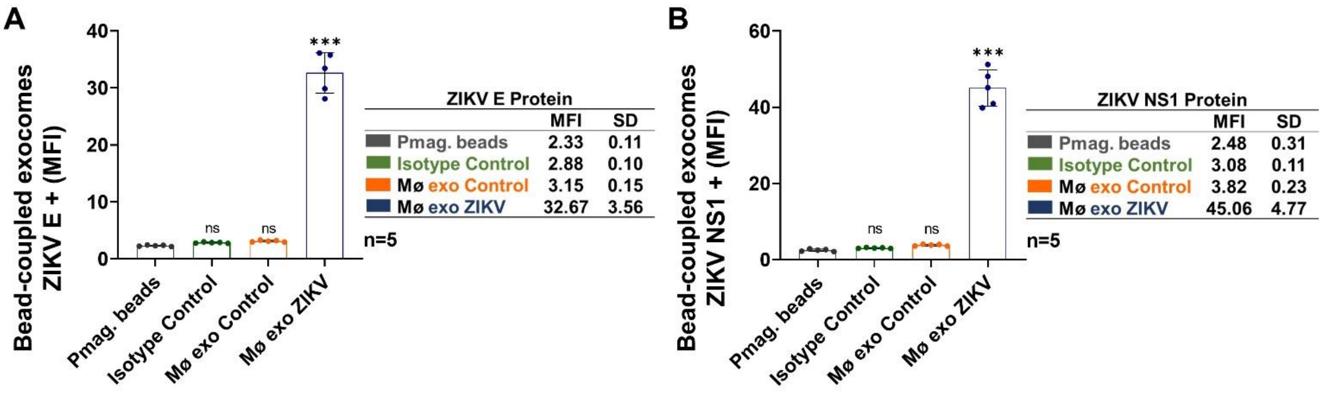

Figure S12. Citofluorometry MFI values from detection of viral E and NS1 proteins in pmag-bead-coupled exosome isolates. (**A**) MFI values of pmag-bead-coupled exosomes ZIKV E+. (**B**) MFI values of pmag-bead-coupled exosomes ZIKV NS1+. The MFI values of ZIKV E and NS1 from pmag-bead-coupled exosomes were compared with the pmag beads values by one-way ANOVA. Statistical significance is denoted as *** when $p < 0.0001$. ns = no significance.

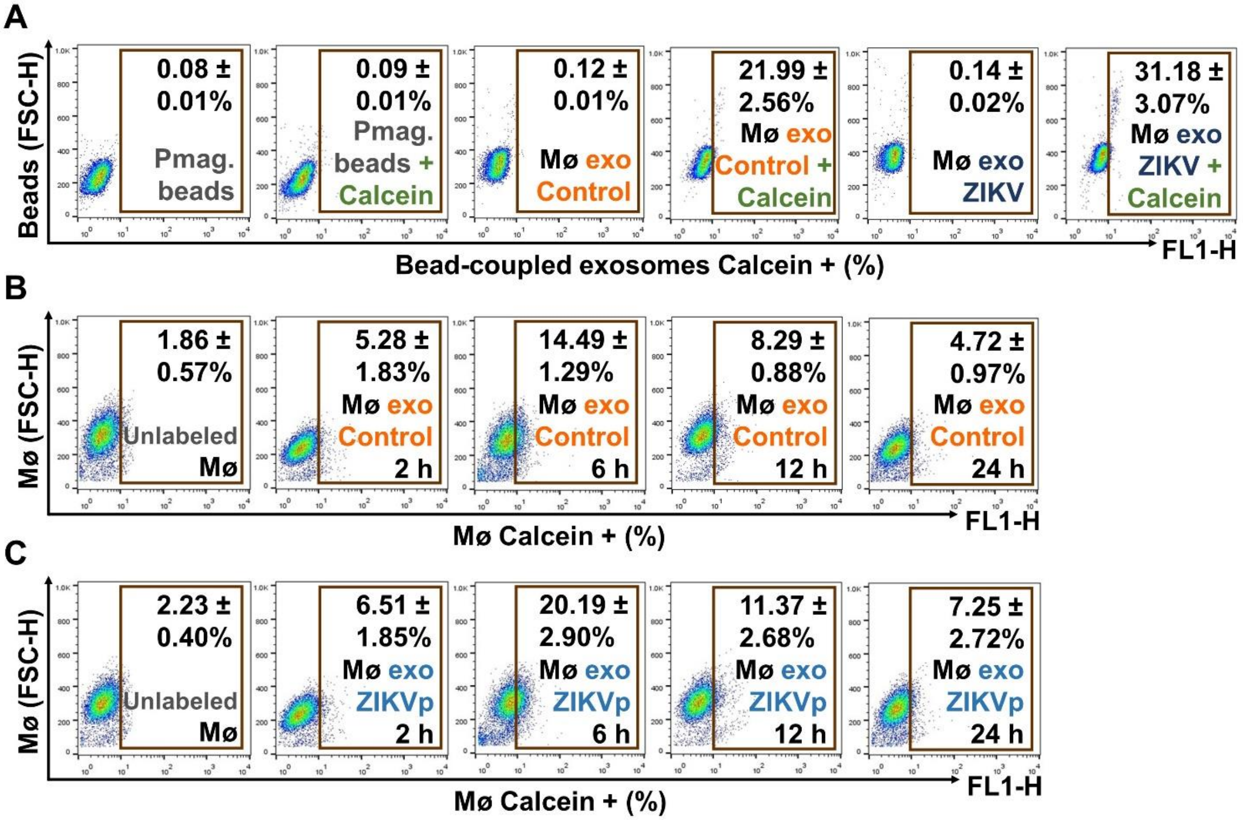

Figure S13. Detection of Calcein AM staining in paramagnetic-beads-coupled exosomes and exosome-stimulated Mø (representative dot plots) by FACS. (**A**) Percentages of pmag-bead-coupled exosomes positive for Calcein AM. (**B**) Detection of exosome (Mø exo Control) and naïve Mø interaction at 2, 6, 12, and 24 h p.i. (**C**) Detection of exosome (Mø exo ZIKVp) and naïve Mø interaction detection at 2, 6, 12, and 24 h p.i. Pmag-beads (dark gray), Unlabeled Mø (light gray), Calcein AM (green), Mø exo Control (orange), and Mø exo ZIKVp (sky blue).

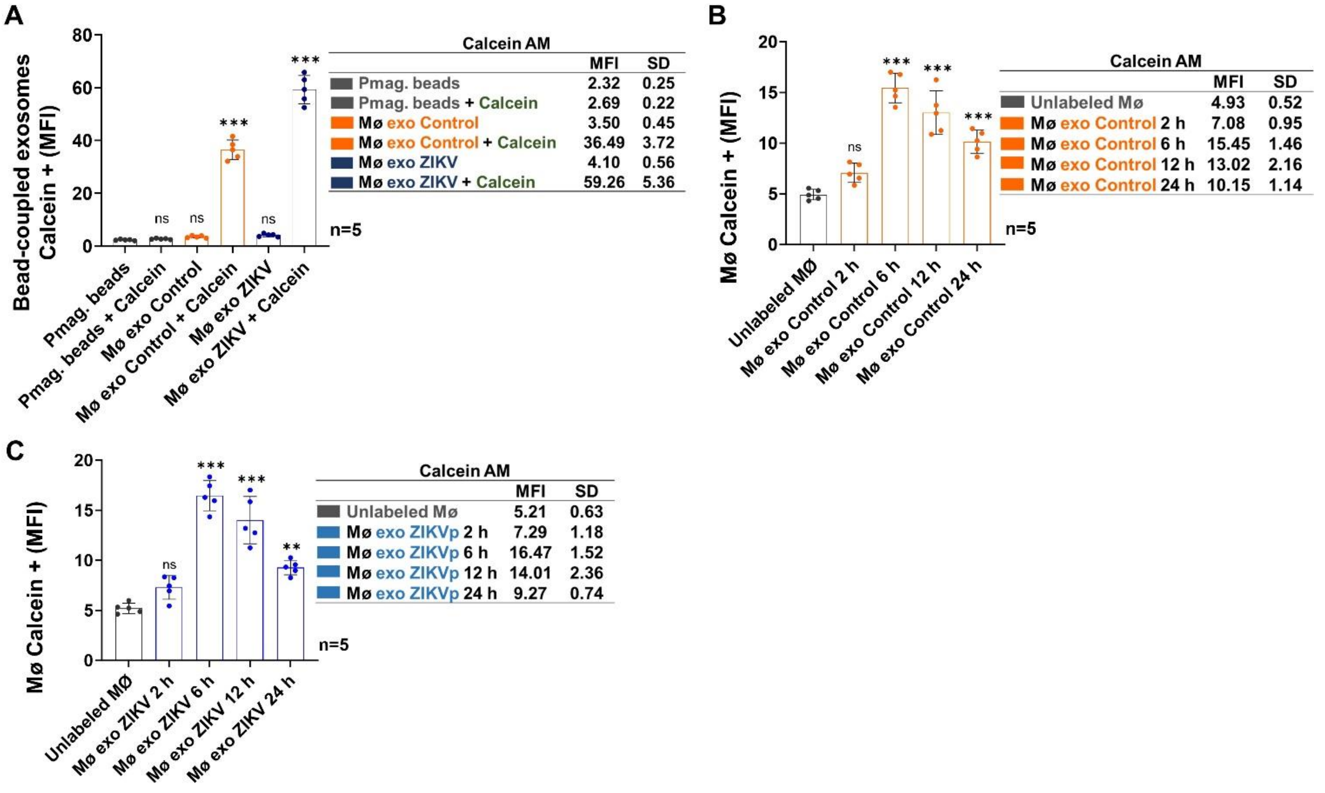

Figure S14. Citofluorometry MFI values obtained by Calcein AM staining in paramagnetic-beads-coupled exosomes and exosome-stimulated Mø. (**A**) MFI values of pmag bead-coupled exosomes Calcein+. (**B**) MFI values of Mø exo Control-stimulated Mø Calcein+. (**C**) MFI values of Mø exo ZIKVp-stimulated Mø Calcein+. The MFI values from pmag bead-coupled exosomes and exosome-stimulated Mø positive for Calcein AM were compared with the control values by one-way ANOVA. Statistical significance is denoted as follows: ** when $p < 0.01$ and *** when $p < 0.0001$. ns = no significance.

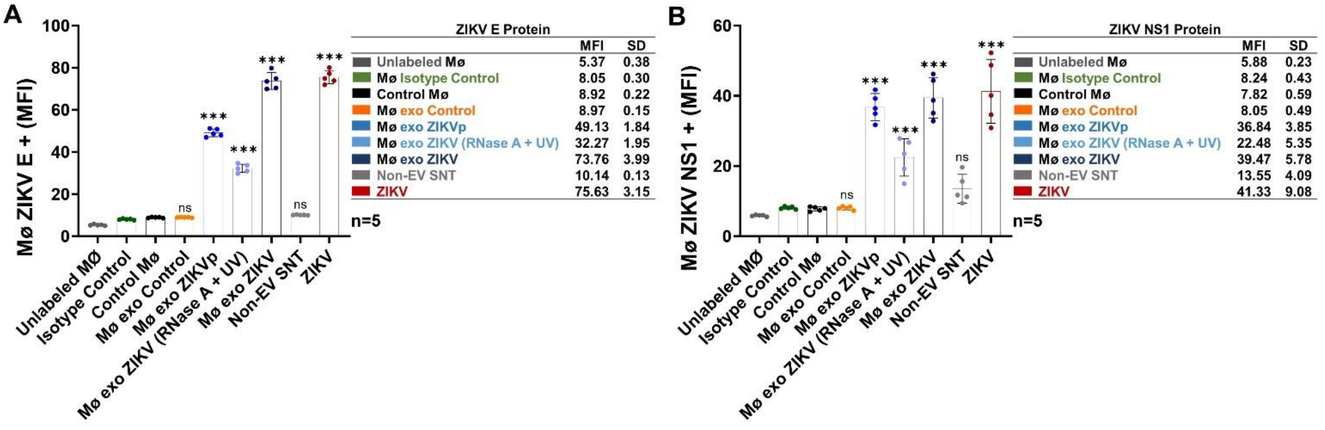

Figure S15. Citofluorometry MFI values from the detection of viral E and NS1 proteins in naïve Mø stimulated with exosome isolates. (**A**) MFI values of Mø ZIKV E+. (**B**) MFI values of Mø ZIKV NS1+. The MFI values of ZIKV E and NS1 from stimulated Mø were compared with the Control values by one-way ANOVA. Statistical significance is denoted as *** when $p < 0.0001$. ns = no significance. Unlabeled Mø (gray), Isotype control (green), Control Mø (black), Mø exo Control (orange), Mø exo ZIKVp (sky blue), Mø exo ZIKV (RNase A + UV) (light blue), Mø exo ZIKV (dark blue), Non-EV SNT (light gray), and ZIKV (red).

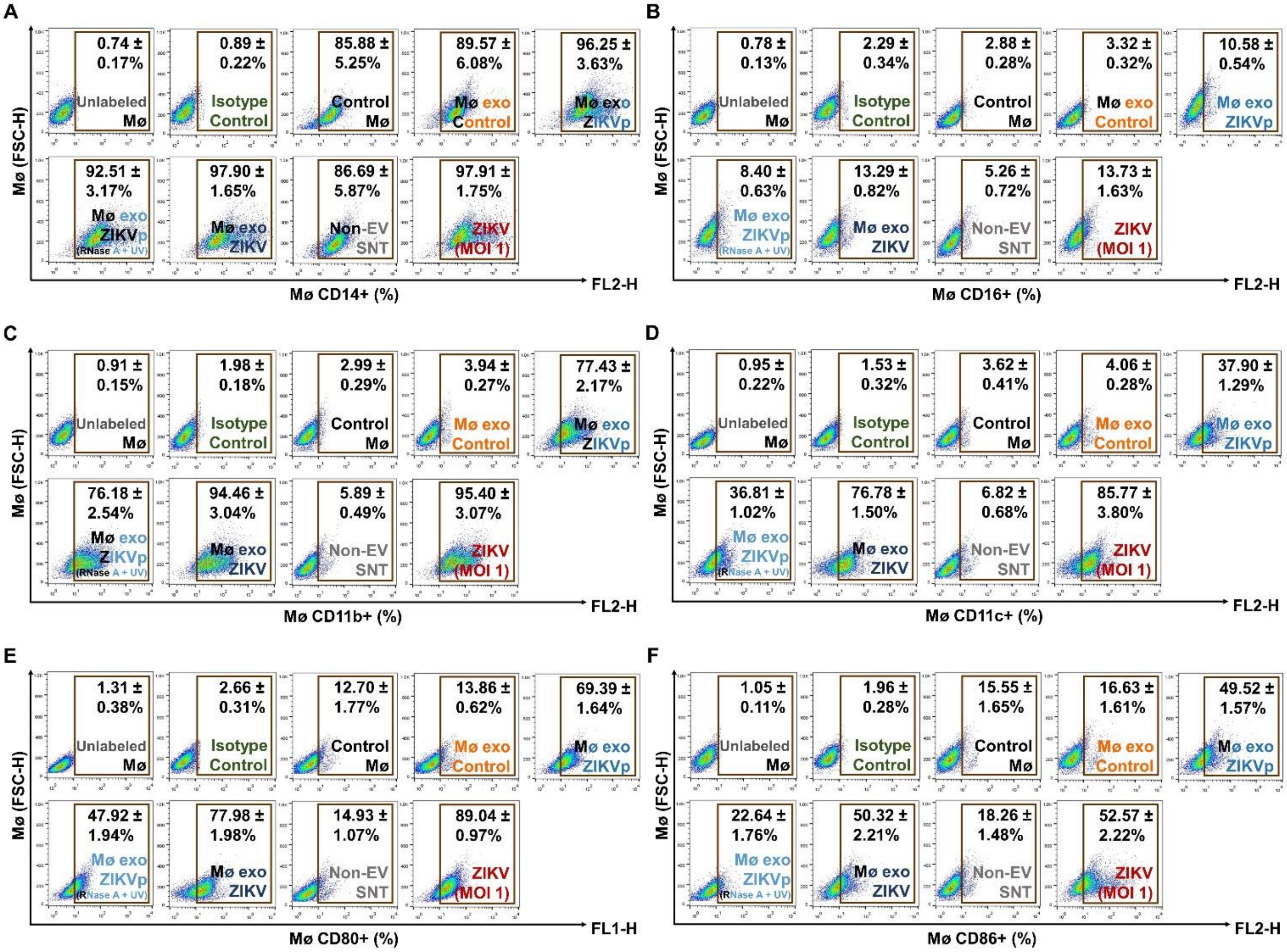

Figure S16. Detection of monocyte (Mø) membrane markers in exosome-stimulated naïve cells (representative dot plots) by FACS. (**A**) Percentages of Mø positive for CD14. (**B**) Percentages of Mø positive for CD16. (**C**) Percentages of Mø positive for CD11b. (**D**) Percentages of Mø positive for CD11c. (**E**) Percentages of Mø positive for CD80. (**F**) Percentages of Mø positive for CD86.

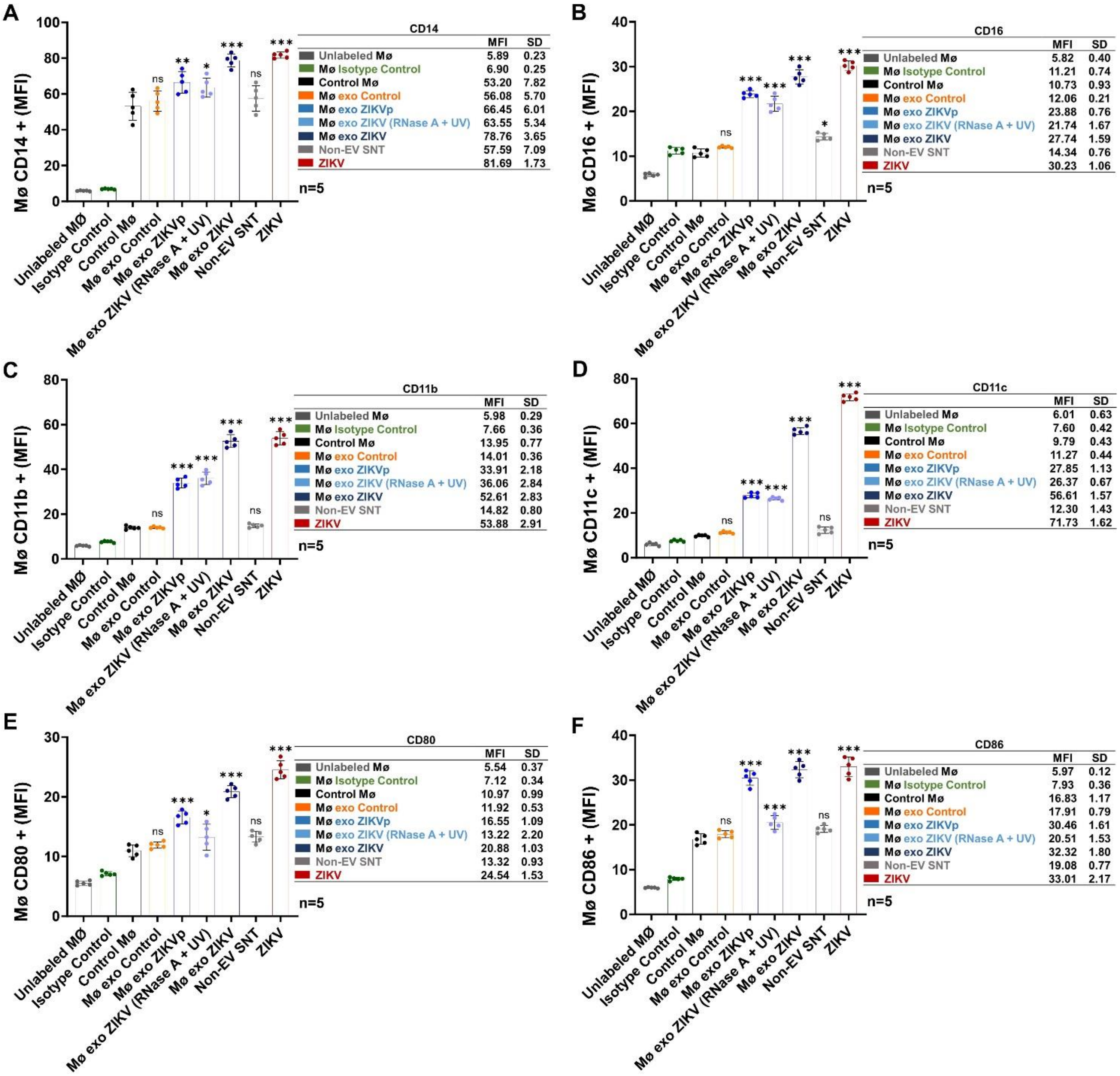

Figure S17. Citofluorometry MFI values from detection of cell markers in naïve Mø stimulated with exosome isolates. (**A**) MFI values of Mø ZIKV CD14+. (**B**) MFI values of Mø ZIKV CD16+. (**C**) MFI values of Mø ZIKV CD11b+. (**D**) MFI values of Mø ZIKV CD11c+. (**E**) MFI values of Mø ZIKV CD80+. (F) MFI values of Mø ZIKV CD86+. The MFI values of CD14, CD16, CD11b, CD11c, CD80, and CD86 from stimulated Mø were compared with the Control values by one-way ANOVA. Statistical significance is denoted as follows: * when $p < 0.05$, ** when $p < 0.01$, and *** when $p < 0.0001$. ns = no significance.
